# The Spectrum of Neuroendocrine Neoplasia: A Practical Approach to Diagnosis, Classification and Therapy by Sylvia L. Asa, Stefano La Rosa, Ozgur Mete

**DOI:** 10.1007/s12022-023-09785-x

**Published:** 2023-08-24

**Authors:** Alessandro Vanoli

**Affiliations:** 1 Department of Molecular Medicine, Unit of Anatomic Pathology, University of Pavia, Pavia, Italy; 2 Unit of Anatomic Pathology, IRCCS San Matteo Hospital Foundation, Pavia, Italy

**Keywords:** Biomarker, Metastasis, Neuroendocrine tumor, Neuroendocrinology

*The Spectrum of Neuroendocrine Neoplasia. A Practical Approach to Diagnosis, Classification and Therapy* [1] by Sylvia L. Asa, Stefano La Rosa, and Ozgur Mete is a book of 478 pages, well-structured in 19 chapters, for pathologists, pathology residents, clinicians, molecular biologists, and all practitioners with interest in the fascinating topics of neuroendocrine pathology and neuroendocrinology (Fig. 1). It is written by a multidisciplinary team of internationally renowned experts in the field of neuroendocrine oncology with a modern and practical approach to neuroendocrine neoplasms.

**Fig. 1:**
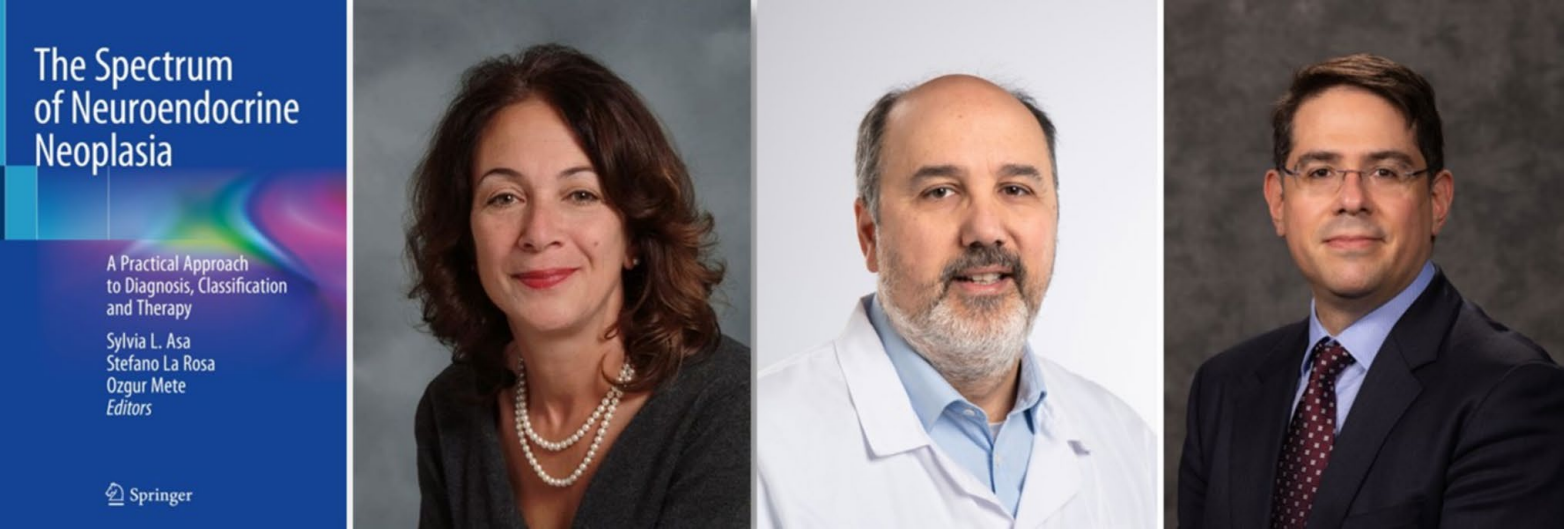
The book cover and book editors Dr. Sylvia L. Asa, Dr. Stefano La Rosa, and Dr. Ozgur Mete, from left to right

The volume begins with an interesting chapter entitled “Neuroendocrine Neoplasms: historical background and terminologies” which offers a nice review of the complex history and evolution of neuroendocrine pathology and related taxonomic terminology within the common classification framework of neuroendocrine neoplasms endorsed by 2022 WHO Classification of Endocrine and Neuroendocrine Tumors [2, 3]. It is followed by two chapters with a clinical perspective dedicated to diagnosis and functional and radiological imaging of neuroendocrine neoplasms. A site-based approach is applied to the central chapters of the book, spanning from the hypothalamus and pituitary, through the upper aerodigestive tract/ear/salivary glands, the thyroid, the parathyroids, the thorax, the gastro-entero-pancreatic system, the genitourinary tract, the breast, the skin, to paragangliomas and metastatic neuroendocrine neoplasms of unknown primary site. Morphologic and molecular features, as well as clinically relevant prognostic and predictive factors, of the diverse types of epithelial and non-epithelial neuroendocrine neoplasms are addressed drawing the spectrum of the heterogeneous family of neuroendocrine neoplasms. The last three chapters are focused on transversal and challenging topics, including the role of cytology in diagnosing neuroendocrine neoplasms, the inherited neuroendocrine neoplasms and, finally, the surgical, medical, and radiation therapeutic management of neuroendocrine neoplasia, including promising future directions.

The book is enriched with a wonderful iconography and several tables which summarize the chapter content and may guide practicing pathologists in differential diagnosis. The clarity and completeness of information of texts, images, and tables are worth of note. The general layout is nice, the Index is complete and useful, and eBook option is unvaluable tool for readers.

In particular, I think that Chapter 16 dedicated to “Metastatic Neuroendocrine Neoplasms of Unknown Primary” is very useful, especially for the thorough overview of the numerous immunohistochemical markers (including transcription factors, hormone markers, etc.), some of which, beyond helping diagnosticians in the detection of occult primary site of a metastatic neuroendocrine neoplasm and in differential diagnosis between histologically similar entities, may also have prognostic value.

Likewise, I appreciated very much the special attention that is given to the inherited neuroendocrine neoplasms in Chapter 18. In fact, hereditary tumor syndromes represent very relevant risk factors to some types of neuroendocrine neoplasms (both paragangliomas and epithelial neuroendocrine tumors and carcinomas), and there is increasing evidence that pathologists may give an actual contribution to suggest an underlying inherited syndromes by identifying, on the microscope, morphologic and/or immunohistochemical harbingers of such predisposing conditions in tumor samples they are examining [4, 5]. The intriguing and related topic of precursor lesions of neuroendocrine neoplasia is also addressed in this chapter, as well as in various other organ-specific book chapters, e.g., in Chapter 7 on “Thyroid Neuroendocrine Neoplasms,” where the C cell hyperplasia is described.

I give it 5 stars and I recommend this book, not only to dedicated endocrine pathologists, but also to all pathologists and clinicians, who may encounter a patient with neuroendocrine neoplasm during their daily routine or may be interested in neuroendocrine research.

## Data Availability

Not applicable.
